# The Influence of Liquid–Solid Preparations on the Dissolution of Suvorexant

**DOI:** 10.3390/polym18080936

**Published:** 2026-04-10

**Authors:** Barbara Jadach, Nikola Pawlak

**Affiliations:** Division of Industrial Pharmacy, Chair and Department of Pharmaceutical Technology, Poznan University of Medical Sciences, 3 Rokietnicka, 60-806 Poznan, Poland

**Keywords:** liquid–solid, suvorexant, dissolution study, enhance dissolution

## Abstract

This study aimed to evaluate the impact of liquid–solid (LS) systems on the dissolution profiles of a poorly soluble drug—suvorexant (SUV). In the first stage of this study, LS systems were prepared by using two different non-volatile solvents: ethylene glycol diethyl ether and polyethylene glycol 400 (PEG 400). To compare the properties of different types of LS systems, formulations were prepared that differed in the content of SUV (10 and 20 mg) as well as in the ratio of excipients (microcrystalline cellulose and colloidal silica), which was 10:1 or 1:1. The physicochemical properties of the prepared formulations were characterized by X-ray diffractometry (XRD), thermogravimetry (TGA) and differential scanning calorimetry (DSC). This was followed by a dissolution study of SUV from prepared LS systems, using a 0.4% sodium lauryl sulfate solution as the medium to maintain sink conditions. Results showed that the LS systems change the crystalline structure of SUV to an amorphous one and improve the dissolution rate of SUV. The greatest improvement was achieved by using the microcrystalline cellulose and colloidal silica in a 10:1 ratio for the preparation of the system (CCA variant). It was observed that the type of solvent used and the order of combining excipients during the preparation of LS systems are also important for the properties. The main point was that physicochemical characterization of the prepared formulations lead to a loss of crystallinity of SUV associated with its introduction into liquid–solid systems.

## 1. Introduction

**Figure 1:**
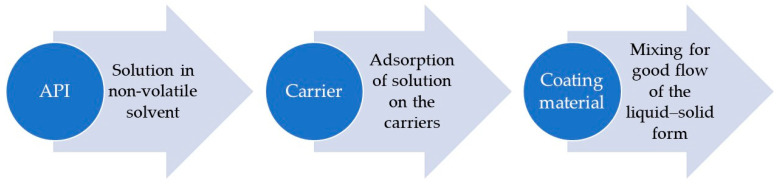
Schematic steps of the preparation of a liquid–solid system.

The lack of solubility or poor solubility of a drug in water is a major limitation for the effective use in practice of both well-known and newly discovered medicinal substances. This is because solubility is the main parameter influencing the achievement of the desired blood concentration by a given medicinal substance, which in turn translates into the therapeutic effect. Additionally, achieving therapeutic concentrations of poorly soluble substances requires the use of large doses, which is often responsible for the increased occurrence of side effects. The desire to use the potential of substances with limited solubility has contributed to the discovery of many techniques that improve their dissolution rate [1]. These include methods such as micritization, lyophilization, salt formation, and solid dispersions, which constitute only a small part of the available options that are used in the pharmaceutical industry [1,2]. One of these techniques with promising results is the creation of liquid–solid (LS) systems [3], which are powdered forms of liquids that are characterized by specific flowability and compressibility as well as apparent dryness. The basis of the technology of these systems is the conversion of a liquid form of a drug, i.e., a solution or suspension of a water-insoluble drug, in a non-volatile solvent, i.e., into a powdered form. This is possible thanks to the use of excipients with appropriate properties, referred to as carriers and coating materials, which, in combination with the liquid form of the drug, allow for the preparation of LS systems [1,2,3]. There are three possible mechanisms that allow liquid–solid systems to increase the solubility of drug substances. These include: increased drug surface area, increased water solubility, and increased wettability. These mechanisms thus improve the dissolution rate, as the solubility of the substance is one of the main factors determining it. They also contribute to improving oral bioavailability. This is due to the fact that the dissolution of a non-polar drug is often the rate-limiting step in absorption in the gastrointestinal tract. Therefore, better bioavailability will be achieved when an orally administered, water-insoluble drug is in solution, thus exhibiting an increased dissolution rate [4,5]. If the drug substance in the LS system is completely dissolved in the solvent, then, in the form of a powder, it is still in a dissolved, molecularly dispersed form. This means the surface area of the drug from which the molecules can be released is larger in the LS system compared to conventional tablets [2,4,6,7,8]. It also follows that with an increase in the amount of a medicinal substance exceeding the solubility limit (i.e., with an increase in the fraction of dissolved drug in the solvent), the release rate will be reduced [2,8]. LS systems, in addition to improving drug release, can be expected to increase the solubility of the drug in water, but a relatively small amount of solvent is not sufficient to increase the overall solubility of the drug substance in water [1,2,8]. However, if the solvent acts as a cosolvent, then it is possible that at the interface of solid particles and the releasing medium, the amount of solvent released from a single particle of the LS system (together with drug molecules) may be sufficient to increase the solubility of the substance in water [2,4,8]. The solvent may act as a surfactant or have a low surface tension, thereby reducing the interfacial tension between the LS particles and the medium in which they are dissolved, thereby improving the wetting properties of the LS particles. To prepare the LS system, a solvent, a carrier, and coating substances are used (Figure 1).

The main solvents which are in use are non-volatile organic solvents, such as polypropylene glycol, glycerin, and polyethylene glycol 200 and 400 (PEG 200 and PEG 400), as well as polysorbate 20 and 80 [1,2,8]. Important properties that should characterize the solvent used are chemical inertness, high boiling point, and low viscosity. Additionally, to ensure penetration into the aqueous environment, the solvent should be miscible with water. The solubility of the drug in the solvent used is also important, as it affects the amount of excipients used and the release profile. The more soluble the drug, the less carrier and coating material will be needed to absorb the solvent and obtain a dry powder. Greater solubility of the drug in the solvent also ensures an increase in the dissolution rate [4,8]. Carriers usually consist of substances with a porous structure and closely arranged fibers, such as various types of cellulose, starch, and lactose [4,8,9,10]. Microcrystalline cellulose is the most commonly used, but substances such as magnesium aluminosilicate and synthetic anhydrous dibasic calcium phosphate are becoming more and more important because they have a greater absorption capacity than microcrystalline cellulose [6,11]. The structure of the carrier makes it have both absorption and adsorption properties, enabling the absorption of the liquid form of the drug [1,11,12]. The coating material covers the surface and maintains appropriate dryness and flowability of the powder during the preparation of LS systems [2,8]. It continues the adsorption process initiated by the carrier, thanks to its hydrophilic fine particles and large specific surface area [1,11]. Colloidal silica is most often used as a coating material, but calcium silicate and magnesium aluminum silicate are also common.

Examples of LS applications described in the literature include examples of the use of the liquid–solid technique to improve the dissolution rate of small doses of insoluble drugs, such as furosemide [13], prednisolone [14], valsartan [15], spironolactone [16], clonazepam [17], naproxen [14,18], tadalafil [19] and raloxifene hydrochloride [20]. In the case of drugs that require higher doses, e.g., carbamazepine, an LS system can be created if we use substances with greater absorption properties, i.e., with a greater ability to absorb liquid using the carrier and the coating material. An example of such a substance is magnesium aluminum silicate, which is characterized by a large specific surface [18,19].

Despite being classified as a BCS class II drug, suvorexant presents formulation challenges that extend beyond low aqueous solubility. SUV is a highly crystalline compound with poor wettability and slow dissolution, which limits its bioavailability and complicates development of solid oral dosage forms. Existing approaches to improve its solubility have focused mainly on salt formation, solid-state modification, and cocrystal engineering. At the same time, no studies to date have explored liquid–solid (LS) systems as a dissolution-enhancing platform for SUV. LS technology has proven effective for other poorly soluble drugs, yet its applicability to SUV remains unexamined. Therefore, investigating LS systems for SUV addresses a clear gap by providing a simple, scalable formulation approach and enabling evaluation of how excipient ratios, solvent selection, and order of addition influence dissolution performance.

Previous studies on liquid–solid systems have demonstrated their usefulness for improving dissolution of various BCS class II drugs, such as meloxicam, piroxicam, candesartan cilexetil, and tadalafil, where enhanced release was attributed to amorphization, improved wettability, and increased surface area. However, none of these studies addressed suvorexant, a compound with distinct solubility challenges and a pronounced crystalline character. Moreover, earlier LS research typically focused on formulation composition or solubility enhancement, whereas the combined influence of solvent type, excipient ratio, and sequence of excipient addition has not been systematically evaluated. The present study therefore provides novelty not only by applying LS technology to SUV for the first time but also by dissecting the contribution of these formulation variables, offering mechanistic insight beyond existing LS reports.

In the presented study, the use of an LS system for suvorexant (SUV) was proposed. It is a substance that is a light gray powder; it belongs to BCS class II, which means that it is characterized by high permeability through biological membranes and low solubility in water (0.117 mg/mL) [21,22]. It is a representative of double orexin receptor antagonists and was the first of this group to be registered for the treatment of insomnia. SUV possess good tolerance and fewer side effects compared to other groups of drugs used in this disease [23]. Proposed LS systems were prepared using two non-volatile solvents, ethylene glycol diethyl ether and polyethylene glycol with a molecular weight of 400, and cellulose and Aerosil as carrier and coating substances, respectively. During the preparation of the LS system, different ratios of the carrier and coating material and the order of their addition for each solvent were analyzed. The physicochemical properties of the prepared formulations were characterized by X-ray diffractometry (XRD), thermogravimetry (TGA), and differential scanning calorimetry (DSC). Also, the dissolution of SUV, which enabled the observation of the influence of LS systems, their composition, and preparation method on the release of SUV, was analyzed.

## 2. Materials and Methods

### 2.1. Materials

The model drug suvorexant (SUV) from Medkoo Biosciences (Durham, NC, USA) was a gift from Zentiva (Prague, Czech Republic) and was used as received. Microcrystalline cellulose EMCOCEL 50 M was purchased free of charge from JRS Pharma (Rosenberg, Germany). Aerosil 200 Pharma was purchased from Evonik Industries AG (Essen, Germany). PEG 400 and ethylene glycol diethyl ether were purchased from Sigma–Aldrich (St. Louis, MO, USA). Sodium dodecyl sulphate (SDS), 0.1 mol/L HCl concentrate for dissolution, and phosphate buffer pH 7.4 concentrate for dissolution were purchased from Avantor Performance Materials S.A (Gliwice, Poland).

### 2.2. Preparation of Liquid–Solid

**Figure 2:**
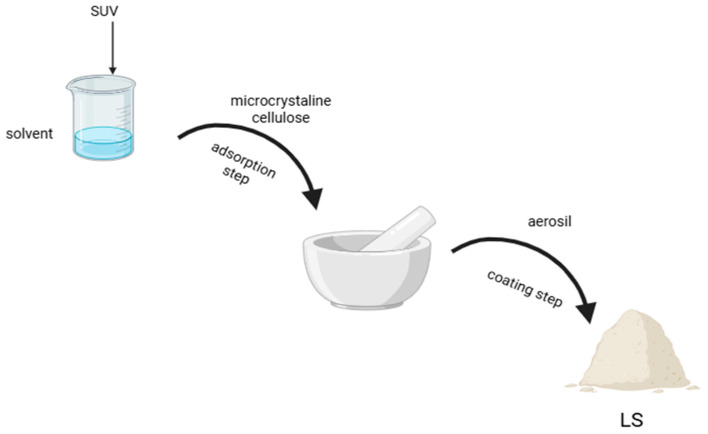
Steps in preparation of LS systems with suvorexant.

In order to obtain LS systems, the drug substance is dissolved or suspended in a non-volatile solvent in an amount that allows the desired concentration to be obtained [1,2]. Then, the appropriate amount of carrier material is added to the liquid medicine in the vaporizer while stirring. Initially, the liquid is captured and absorbed by the internal structure of the carrier. When this process becomes saturated, adsorption occurs on the outer and inner surfaces of the porous carrier particles. The adsorption process is continued by the coating material, which allows a dry powder with appropriate flow properties to be obtained [11]. In the presented study, LS systems with SUV were prepared in accordance with these steps, which are presented in Figure 2.

Briefly, in all formulations, microcrystalline cellulose 50 M was used as a carrier and colloidal silica (Aerosil 200 Pharma) as a coating material. The obtained systems differed in the content of SUV (10 mg or 20 mg), the order of addition and the amount of auxiliary substances, as well as the solvents used, which were ethylene glycol diethyl ether or PEG 400. The differences in the preparation of individual formulations were intended to check which of the combinations used allowed for the best improvement in the dissolution of the poorly soluble SUV and to check for the existence of possible dependencies between the composition or preparation of a given LS system and the amount of SUV released. In later parts of the work, the abbreviations CCA, CA and AC are used to describe the prepared formulations, differing in the order of adding auxiliary substances and in composition, which are presented in Table 1. The CCA formulations, differing in composition from the CA and AC variants, contained microcrystalline cellulose and colloidal silica in a ratio of 10:1. However, CA and AC formulations had the same composition (0.5 g of microcrystalline cellulose and 0.5 g of colloidal silica; 1:1 ratio) but a different order of adding excipients. The exact composition of each of the formulations and the designations used for them are presented in Table 1.

In addition to the combinations of ingredients forming LS systems, solvent-free combinations were also prepared and used as a reference sample in the dissolution study. They were prepared similarly to the other formulations, omitting the SUV dissolution step. The ingredients were ground in a mortar in the appropriate order to obtain uniform, dry powders.

### 2.3. Characterization of Physicochemical Properties

In order to characterize the physicochemical properties of the obtained LS systems, thermogravimetric analysis, differential scanning calorimetry, and X-ray diffractometry were performed. Each of the dry substances included in the LS (suvorexant, microcrystalline cellulose, and colloidal silica) were also analyzed.

#### 2.3.1. X-Ray Diffractometry (XRD)

Each of the tested samples were placed in a holder, tamped to obtain a uniform surface, and inserted into a diffractometer (Phaser D2 Bruker; Billerica, MA, USA). During the study, the 2Theta measurement range was used from 5° to 45° with a step of 0.02° and a counting time of 1 s/step. The diffractometer operated at a voltage of 30 kV and an intensity of 10 mA.

#### 2.3.2. Thermogravimetry (TGA)

Thermogravimetric analysis was carried out in a thermogravimeter (TG 209 F3 Tarsus Netzsch; Weimar, Germany), in which crucibles containing the tested samples were placed and weighed at an amount of 10.60–24.26 mg (due to its volume, colloidal silica was weighed at an amount of 4.36 mg). An empty crucible was used as a reference test. The test was carried out at a temperature range from 25 °C to 400 °C with a heating rate of 10 K/min and a nitrogen flow of 30 mL/min.

#### 2.3.3. Differential Scanning Calorimetry (DSC)

Samples were weighed at an amount of 5.13–9.68 mg (due to its volume, colloidal silica was weighed at an amount of 2.05 mg) and placed in tightly closed aluminum crucibles, which were then transferred to a differential scanning calorimeter (DSC 214 Polyma Netzsch; Weimar, Germany) and tested at a temperature range from 25 °C to 300 °C with a constant temperature increase rate of 10 K/min and a nitrogen flow of 30 mL/min. An empty aluminum crucible was used as a reference test.

### 2.4. Dissolution Studies of SUV from Prepared Liquid–Solid Systems

The dissolution studies of SUV from the prepared LS systems, as well as from reference samples without solvent, were performed in a paddle apparatus (DT 126 light Erweka; Hessen, Germany). The test medium used was a 0.4% SDS solution to maintain sink conditions. The test was carried out in 300 mL of medium for each series (*n* = 3) at a temperature of 37 °C for 120 min. The rotation speed of the paddles was set to 75 rpm. For the test samples, the powder was used at an amount corresponding to the content of 2 mg of SUV. At specific time points, 5, 15, 30, 45, 60, 90, and 120 min, samples with a volume of 4 mL were taken and filtered through membrane filters with a pore diameter of 0.20 μm. After each collection, the chambers were filled with the appropriate amount of fresh SDS solution. The amount of released SUV was determined using a validated UV–Vis spectrophotometric method. The concentration of SUV in the collected samples was determined using a validated UV–Vis spectrophotometric method. Measurements were performed at λ = 254 nm, corresponding to the absorption maximum of SUV in 0.4% SDS solution. Quantification was based on an external calibration curve prepared in the same medium, with a linearity range of 2–20 µg/mL and a correlation coefficient of R^2^ > 0.999. Method repeatability (expressed as RSD) was below 2%, and recovery values ranged from 98 to 102%, confirming accuracy. Blank samples of the dissolution medium were used as a reference. This method is routinely applied in our laboratory for SUV quantification and was deemed suitable for the present dissolution study. Results are presented as cumulative release profiles. The obtained SUV release profiles from the prepared solid–liquid formulations were used to assess their similarity using the similarity factor f_2_ [24,25]. The statistical method, independent of the model used for comparison, was established using the DDSolver program. The following equation [24,25] was used in the calculations (1), where f_2_ is the similarity coefficient, R_t_; T_t_ is the % dissolved/released API for the evaluated sample and reference sample at time point t; n is the number of samples; and t is the time point:

(1) $$f_{2}={{50\times x\log\{[1+\frac{1}{n}\sum_{i=1}^{n}(R_{t}-T_{t})}^{2}]}^{-0.5}\times 100\}.$$

### 2.5. Statistical Analysis

The data were analyzed using Statistica software ver. 13 (TIBCO Software Inc., Palo Alto, CA, USA). Analysis of variance (ANOVA) was used to determine the statistical significance between samples. The a priori level of significance was *p* < 0.05. The experiments were completed in triplicate (*n* = 3), and the results are represented as mean ± standard deviation (SD).

## 3. Results and Discussion

The solubility of a drug substance is a key factor determining the dissolution of the drug from the prepared system. Before the preparation of LS systems, a qualitative solubility screening was performed to verify whether SUV could be dissolved or uniformly dispersed in the selected non-volatile solvents (ethylene glycol diethyl ether and PEG 400). This preliminary step aimed to confirm the feasibility of incorporating SUV into the liquid phase of the LS systems rather than to generate quantitative solubility values. Both solvents demonstrated sufficient capacity to dissolve SUV at the concentrations required for LS preparation, and therefore detailed numerical solubility measurements were not conducted.

One of the ways to improve the dissolution rate of poorly soluble substances is to obtain liquid–solid systems. Non-volatile solvents are used in their preparation, and the solubility of SUV in the solvents used in the study is assessed. During the presented study, 12 different LS formulations were prepared, differing in the solvent used, SUV content, and amount and order of excipients. For each of the prepared formulations, physicochemical characteristics and a dissolution study were performed in order to assess the impact of LS systems on the above aspects and to compare the results with each other.

### 3.1. Physicochemical Characteristics of Prepared Liquid–Solid Systems

#### 3.1.1. X-Ray Diffractometry (XRD) of LS Systems

**Figure 3:**
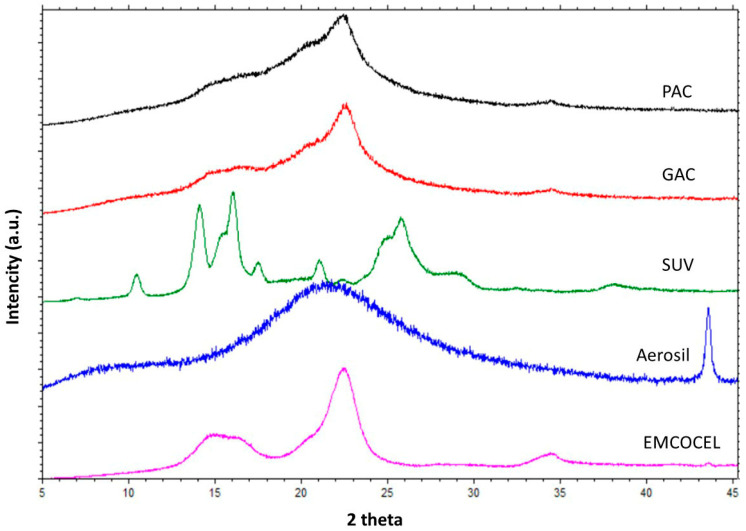
XRD diffractograms of ingredients used for preparation of chosen LS systems.

During the study, diffractograms of all obtained LS systems and dry components were evaluated. Diffraction patterns of substances included in the LS systems (SUV, microcrystalline cellulose, and Aerosil) are shown in Figure 3.

The broad diffraction peaks observed in the diffraction pattern of colloidal silica indicate its amorphous structure, while the narrow diffraction peaks of SUV confirm its crystalline structure. In the diffraction pattern of SUV, three characteristic peaks can be observed whose angular positions are similar to those obtained by Gundlapalli et al. [21] for two crystal forms of suvorexant (SUV form I and SUV form II). A different situation can be observed in the case of microcrystalline cellulose, as cellulose chains contain both crystalline (ordered) and amorphous (disordered) regions [26]. This fact is confirmed by the obtained diffractogram, which shows both components of the substance. The amorphous part (amorphous hump) can be observed in the range of 14.5–16.5° (2θ measurement range), followed by a sharp peak at 2θ = 22.5°, indicating the crystalline structure of the substance, and probably a small amorphous peak at approximately 2θ = 34.5°. A similar diffraction pattern of microcrystalline cellulose was obtained by Hussain et al. [27] and Lu et al. [19], who obtained a diffractogram with a crystalline peak of the highest intensity at 2θ = 22.5° and an amorphous part located at approximately 2θ = 18°, which corresponds to the angular positions of the characteristic peaks obtained in this study.

Peaks characteristic of SUV were not observed in the diffractograms of LS systems (those selected are presented in Figure 3), in which ethylene glycol diethyl ether and PEG 400 were used as the solvents. This may indicate the transformation of the crystalline form of the drug into an amorphous one. The peaks visible in the diffractograms of LS systems are consistent with the diffractogram of microcrystalline cellulose, the most characteristic of which is the peak at 2θ = 22.5°. These peaks differ in intensity, which is related to the content of microcrystalline cellulose in the formulation. The highest intensity, around 8000, was achieved by CCA formulations with the highest cellulose content (approx. 1.0 g). However, CA/AC formulations with a lower content of this ingredient (approx. 0.5 g) achieved lower peak intensity, ranging from approx. 3600 to 4400. In similar studies conducted by Dias et al. [3], using LS systems with a different active substance (meloxicam), and for systems with piroxicam obtained in the study conducted by Jabbar et al. [28], on the diffractograms, no peaks characteristic of the active substances used were observed. This indicates that during the preparation of LS systems, only microcrystalline cellulose retains its crystalline state, while the active substance loses its crystallinity, as evidenced by the lack of peaks characteristic of the active substance.

In the present study, quantitative determination of the degree of crystallinity was not performed because the composition of the LS systems makes such analysis unreliable and of limited interpretative value. SUV is present in relatively low amounts compared with the bulk of microcrystalline cellulose and colloidal silica, whose signals dominate the diffractograms. After incorporation into the LS systems, the characteristic peaks of crystalline SUV disappear completely, indicating its transition to an amorphous or molecularly dispersed form. Under these conditions, numerical deconvolution of crystalline and amorphous contributions would not accurately reflect the state of SUV, as the excipients overshadow its diffraction pattern. Therefore, a qualitative interpretation based on the loss of SUV peaks, supported by parallel DSC results, is sufficient to confirm amorphization, which is consistent with standard practice in LS system characterization.

Although XRD, DSC, and TGA provide consistent evidence of partial or apparent amorphization of SUV, these techniques cannot fully reveal the molecular nature of potential drug–excipient interactions. Spectroscopic methods such as FTIR or solid-state NMR would allow for detection of specific interactions within the LS matrix; however, they were not included in the current experimental scope, which was limited to screening the effect of formulation variables on dissolution behavior. Such analyses would be valuable in future studies aimed at detailed structural elucidation of LS systems.

#### 3.1.2. Thermal Analysis of LS Systems

**Figure 4:**
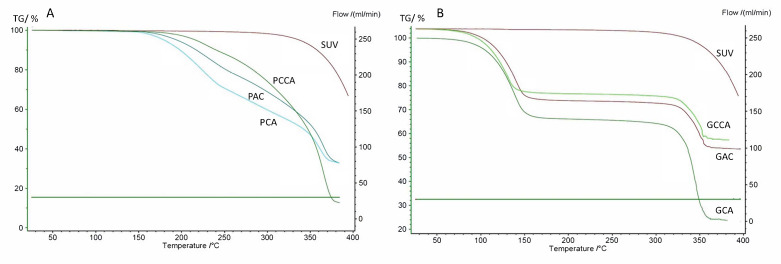
TGA thermograms of SUV and LS systems: (**A**) series prepared with PEG 400; (**B**) series prepared with ethylene glycol diethyl ether.

The results of the thermogravimetric analysis (Figure 4) are presented as the dependence of mass loss (%) on temp. (°C). The TGA thermogram of SUV (results not presented) allows for the observation of mass loss, indicating the degradation of the substance, initiated at approximately 270 °C. Above 300 °C, faster weight loss is observed, progressing to a temperature of 400 °C or the maximum value of the set temp. range.

In turn, the TGA thermogram of microcrystalline cellulose shows a marginal mass loss up to a temperature of 300 °C, which may be related to the evaporation of water associated with the polysaccharide. However, in the range of 300–350 °C, significant weight loss is observed, indicating the decomposition of the compound [29]. The flat TGA thermogram of Aerosil indicates stability in the temp. range used. The TGA results allow the same thermogram to be observed for LS systems in which ethylene glycol diethyl ether was used as a solvent, both for formulations containing 10 mg and 20 mg of SUV (Figure 4B). The weight loss in the temp. range of 320–360 °C is related to the decomposition of microcrystalline cellulose contained in the formulations, as a similar change can be observed in the TGA thermogram of the pure substance. The more intense weight loss for the GCCA formulation is probably related to the higher content of microcrystalline cellulose than in other formulation variants. The mass change observed in the range of 100–150 °C is probably related to the evaporation of the ethylene glycol diethyl ether used from the LS systems, due to the similar boiling point of this solvent, which is 189 °C [30]. TGA thermograms for LS systems in which PEG 400 was used as a solvent (Figure 4A) were similar for both types of formulations, containing 10 mg and 20 mg of SUV. The weight loss observed above 200 °C is probably related to the evaporation of the solvent used, as its boiling point is ˃250 °C [31]. However, the increased weight loss at a temp. of 350 °C results from the degradation of the cellulose contained in the formulation microcrystalline. As in the case of LS systems with ethylene glycol diethyl ether, the increased intensity of mass loss for the PCCA formulation results from the higher content of microcrystalline cellulose in this variant of LS systems.

While the observed mass-loss events in the LS systems occur within temperature ranges close to the reported boiling points of the solvents, these changes should not be interpreted as direct or exclusive indicators of solvent evaporation. In TGA measurements, thermal events may be influenced by several overlapping processes, including desorption of solvent molecules bound within the porous structure of microcrystalline cellulose, release of solvent associated with the excipient surface, partial thermal degradation of solvent–excipient adducts, and interactions affecting the volatility of the components. Therefore, the correspondence between mass loss and the nominal boiling point should be viewed as approximate rather than absolute. The interpretation provided in this study reflects this broader context and acknowledges that complex matrix–solvent interactions can shift or broaden thermal transitions. Quantification of residual solvent content was not performed in this study. The LS systems prepared here contain non-volatile solvents that are primarily retained within the porous carrier or adsorbed onto the surface of excipients rather than present as freely evaporating fractions. Because the thermal mass-loss profile of the formulations is dominated by the decomposition of microcrystalline cellulose and desorption processes from excipient surfaces, TGA does not allow for reliable isolation of a distinct solvent-related mass-loss event suitable for quantification. For this reason, the TGA results were used only to qualitatively characterize thermal behavior and not to determine the exact residual solvent content. This approach is consistent with the exploratory and screening-focused scope of the present study.

#### 3.1.3. Differential Scanning Calorimetry (DSC) of Prepared LS Systems

**Figure 5:**
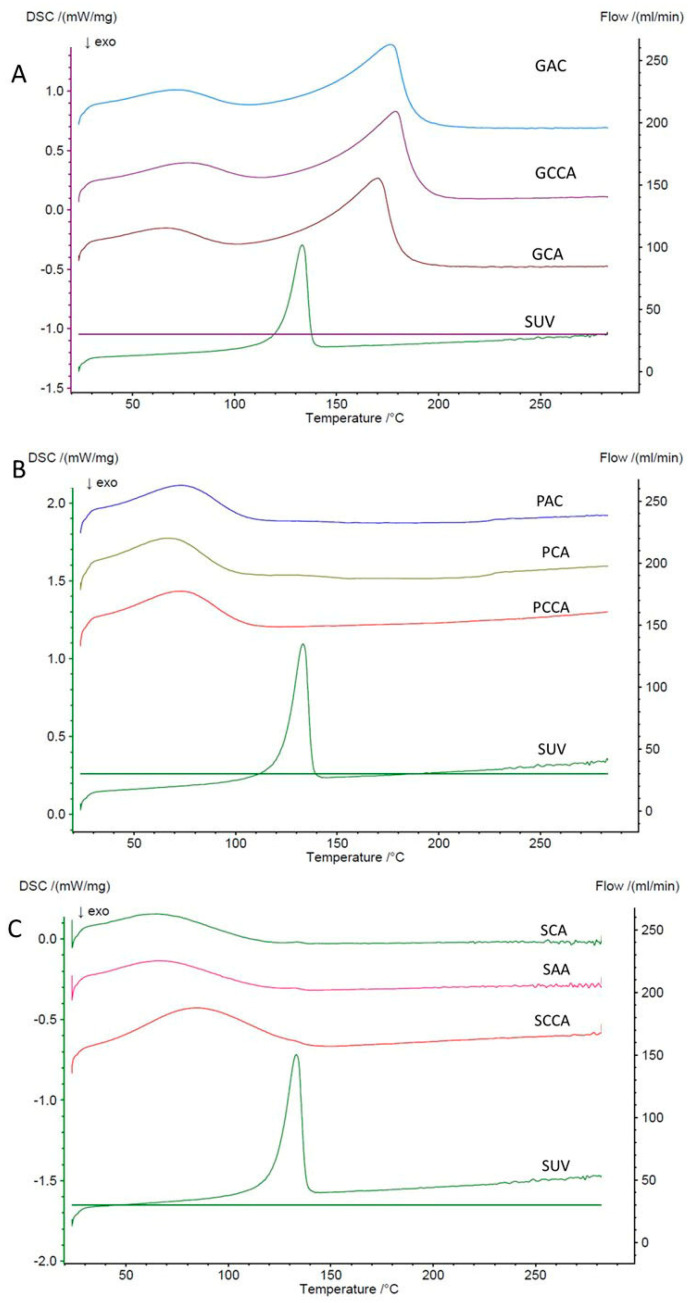
DSC thermograms of LS systems: (**A**) series with ethylene glycol diethyl ether used as solvent; (**B**) series with PEG 400 used as solvent; (**C**) systems prepared without solvent.

Differential scanning calorimetry was performed to determine possible changes in the thermal behavior of the drug related to its introduction into the LS system. DSC was used to characterize the dry components of LS systems (Figure 5C) using two solvents: ethylene glycol diethyl ether (Figure 5A) and PEG 400 (Figure 5B).

The DSC thermogram of SUV shows a sharp endothermic peak at 132.9 °C, associated with the melting of the drug [32]. This proves the crystalline form of pure SUV. However, the endothermic, broad peak in the range of approximately 45–140 °C with a maximum at 83.7 °C in the DSC thermogram of microcrystalline cellulose is associated with the evaporation of water from the polysaccharide [33,34]. This confirms the marginal water loss visible in the TG thermogram of this compound. The flat course of the DSC curve of colloidal silica indicates the lack of significant thermal transformations occurring in its substance during analysis in the applied temp. range of 25–300 °C, which can also be observed in the TGA thermogram of this compound. The DSC thermogram of LS systems with ethylene glycol diethyl ether as the solvent, both for formulations using 10 mg and 20 mg of SUV, showed the lack of a characteristic peak for SUV, bound with its melting point, which was observed for the pure substance.

In the range of approximately 35–100 °C, an endothermic peak is visible related to the evaporation of water from microcrystalline cellulose, which also appears in the thermogram of the pure compound. Peaks whose maxima occur in the range of 165–180 °C are probably related to the evaporation of the solvent, whose boiling point is 189 °C [30]. This change is also visible in the TGA thermogram for LS systems with ethylene glycol diethyl ether in Figure 4. In the thermogram of LS systems in which PEG 400 was used as a solvent, for both formulations in which 10 mg and 20 mg of SUV were used, similarly to the case of using ethylene glycol diethyl ether, no characteristic peak for SUV, associated with its melting point, was observed, but it was observed for the pure substance [21]. The endothermic peak visible in the thermogram in the range of 35–100 °C is related to the evaporation of water from microcrystalline cellulose [33,34]. The change in the course of the DSC curve, which can be observed from approximately 220 °C, is probably related to the evaporation of the solvent, which is also confirmed by the TG thermogram for LS systems in which PEG 400 was used as the solvent (Figure 4). When analyzing LS systems using differential scanning calorimetry, the disappearance of peaks characteristic of a given active substance is observed. This was observed, among others, during the studies by Kamble et al. [35] on LS systems with rosuvastatin, as well as during the studies conducted by Sayyad et al. [36], in which candesartan cilexetil was used as the active substance and in LS systems with tadalafil obtained by Lu et al. [19]. In all mentioned LS systems, this disappearance of characteristic peaks may indicate the formation of a drug solution in LS systems, i.e., its molecular dispersion in the solid matrix and the loss of crystallinity of the active substance. According to Maur et al. [37], such suppression of the drug’s thermal properties, manifested by the disappearance of peaks, indicates its amorphization.

### 3.2. Assessment of the Impact of Prepared Liquid–Solid Systems on the Dissolution of SUV

For each of the prepared formulations, a dissolution test was performed in a paddle apparatus, using a 0.4% sodium lauryl sulfate solution as a medium. The concentration of SDS used in the dissolution medium (0.4% *w*/*v*) was substantially higher than the reported critical micelle concentration (CMC) of SDS, which is approximately 0.03% *w*/*v* under physiological temperature conditions. At concentrations above the CMC, SDS forms micelles capable of incorporating hydrophobic molecules, thereby increasing the apparent solubility of poorly soluble drugs. In this study, operating well above the CMC ensured adequate micellar solubilization to maintain sink conditions for SUV, which is essential for comparative dissolution testing of BCS class II compounds. The purpose of the SDS concentration was therefore not to mimic physiological environments but to provide a robust, discriminatory release medium allowing for evaluation of formulation-dependent effects.

**Figure 6:**
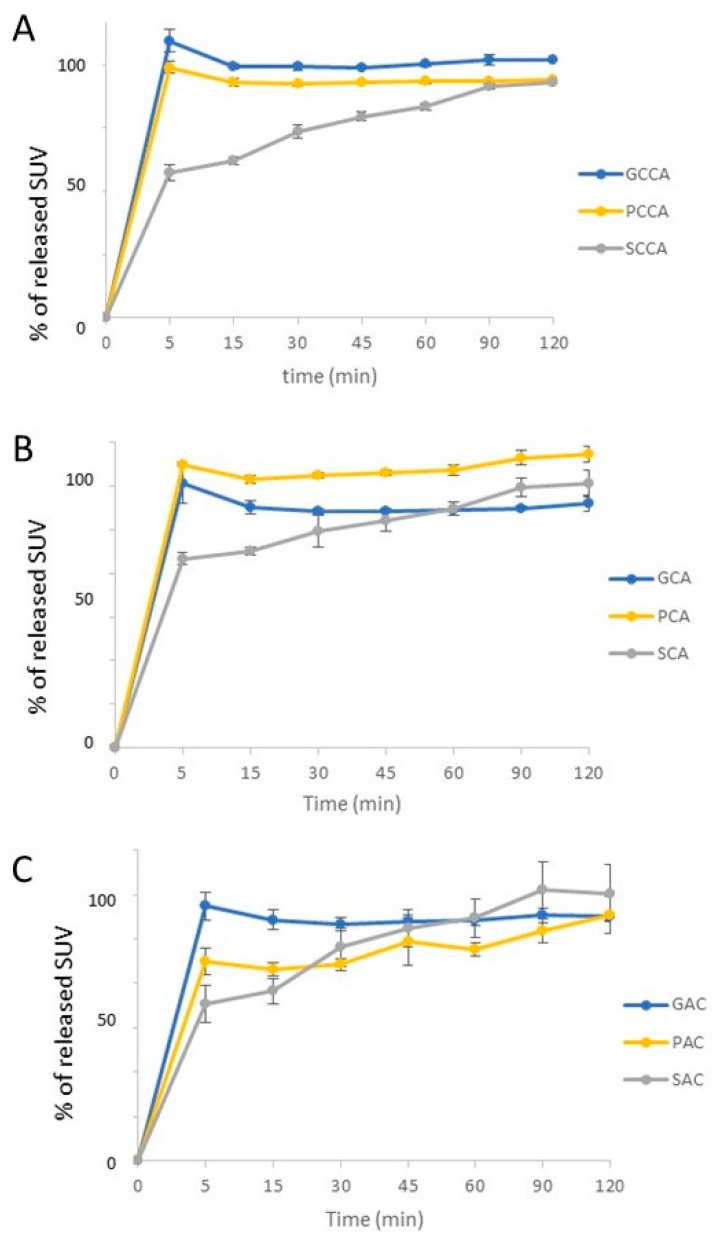
Comparison of release profiles of prepared LS systems and preparation without solvent (series with 10 mg SUV); (**A**) series prepared as CCA versions; (**B**) series prepared as CA versions; (**C**) series prepared as AC versions.

The release rate was tested for LS systems, as well as formulations with the same composition as liquid–solid systems but without solvent (SAC, SCA, and SCCA), whose release profile was used as a reference sample, enabling comparison with the release profiles of prepared LS systems. This allowed us to observe the influence of the solvents used, ethylene glycol diethyl ether and PEG 400, on the dissolution of SUV from the prepared systems. Additionally, it was observed whether changing the ratio of excipients (CCA and CA/AC formulations) and the order of their addition (AC and CA formulations) affects the release of SUV from LS systems, and whether the content of the drug substance (CCA, CA and AC, and CCA2, CA2 and AC2, respectively) changes its release profile. The comparison of selected release profiles is presented in Figure 6.

Analyzing the data obtained from the dissolution study, it was observed that for the first 15 min of the test, a larger amount of SUV was released for almost all prepared LS systems compared to the reference tests in which no solvent was used. These results confirm the effectiveness of LS systems in improving the dissolution rate of sparingly soluble SUV. Comparative analysis using the similarity coefficient *f*_2_ also proves the effectiveness of LS systems in modifying the release/dissolution profile of SUV, as no similarity (*f*_2_ < 50) was demonstrated between the formulations without solvent and all prepared LS systems with the same composition and preparation method but using a solvent (Table 2).

For the reference tests (SCCA, SCA, and SAC), the amount of released SUV increased with the passage of time and reached the highest values at the end time point (after 120 min), while for LS systems, after 5 min of the experiment, full dissolution of the dose was observed. All comparison results are presented in the Supplementary Materials (Table S1).

For formulations containing excipients in a ratio of 1:10 (PCCA and PCCA2), the release profile was similar to the series with ethylene glycol diethyl ether. CCA variants containing different solvents, regardless of the amount of SUV used, had similar release profiles (*f*_2_ > 50). Differences were noticed for the AC and CA variants, for which the amount of SUV released during the study changed slightly (increase/decrease) to reach the highest values at the last time point.

These series in the variant with ethylene glycol diethyl ether did not show any similarity to those in which PEG 400 was used as a solvent. The factor that distinguishes CA/AC formulations from CCA and may influence the obtained result is the mutual ratio of excipients. In addition, the higher solubility of the substance in ethylene glycol diethyl ether compared to PEG 400 may be important. When comparing the results for LS systems differing in the non-volatile solvent used and containing 20 mg of the active substance, larger amounts of released SUV were noticed in the formulations in which ethylene glycol diethyl ether was used as a solvent, compared to those in which PEG 400 was used; this was not noticed in series containing 10 mg of SUV. The obtained release profiles for most LS systems differing in SUV content were similar, with the exception of PCA and PCA2 and GAC and GAC2, which indicates that the use of a larger amount of SUV does not affect the amount of substance released from LS systems. Also, results may indicate the probable importance of the order of adding excipients. When comparing CCA variants with a 1:10 coating material-to-carrier ratio with CA/AC with a 1:1 excipient ratio, no significant relationship was observed with the increase in the amount of SUV released in the ethylene glycol diethyl ether variant, for which most of the release profiles were similar. However, in the case of LS systems with PEG 400 used as a solvent, it was observed that for both formulations containing 10 mg and 20 mg of SUV, the formulations with the higher amount of substance released were those in which cellulose (PCA and PCCA/PCCA2) was added as the first ingredient. This allows us to draw a conclusion about the probable importance of the order of adding excipients in the preparation of LS systems prepared with PEG 400. Support for this relationship can be seen by comparing PCA and PAC formulations, differing only in the order of adding microcrystalline cellulose and colloidal silica: in the PCA series, the amount of released SUV was higher at each time point, and when comparing the *f*_2_ coefficients, no similarity was shown. The existence of a relationship between the order of combining the carrier and the coating material when preparing LS systems when PEG 400 was used as a solvent, and the lack of this relationship when ethylene glycol diethyl ether was used, proves the probable influence of the type of solvent used on the importance of the order of adding auxiliary substances. The effectiveness of liquid–solid systems in improving the dissolution rate of a BCS class II substance (tadalafil) has been proven by Lu et al. [19]. The study used the same medium (aqueous sodium lauryl sulfate) and a similar methodology, but the LS systems with tadalafil were converted into tablets. An improvement in the dissolution rate of the active substance was achieved compared to the pure substance and also to directly compressed tablets containing a mixture of tadalafil with excipients without a liquid carrier. During the analysis of the results, an increase in the amount of drug released was also observed in batches with a higher ratio of excipients, i.e., with a higher content of microcrystalline cellulose.

Given that almost all LS formulations demonstrated an immediate and nearly complete release of SUV within the first few minutes, additional comparative dissolution metrics were not informative. Dissolution efficiency values approached their theoretical maximum and therefore offered no discriminatory power between formulations. Likewise, kinetic modeling could not be meaningfully applied, as the release profiles did not exhibit a time-dependent dissolution pattern that could be fitted to conventional kinetic models. For rapidly dissolving systems such as those obtained here, the similarity factor f_2_ remains the most appropriate tool for assessing differences relative to solvent-free controls, and this parameter was therefore used as the basis for comparative analysis [24,25].

The enhanced dissolution of SUV observed for the LS systems can be attributed to several complementary mechanisms, among which amorphization of the drug appears to play the predominant role. The XRD and DSC analyses demonstrated the absence of characteristic crystalline peaks of SUV, indicating that incorporation into the LS systems leads to its molecular dispersion within the carrier matrix. As reported for other BCS class II drugs, such loss of crystallinity markedly increases the dissolution rate due to the higher free energy and improved thermodynamic solubility of the amorphous form. In addition to amorphization, the LS systems promote dissolution through improved wettability and increased effective surface area [3,19]. The hydrophilic nature and porous structure of microcrystalline cellulose facilitate rapid penetration of the dissolution medium, while the presence of non-volatile solvents contributes to better dispersion of SUV on the carrier surface. These factors allow for immediate medium–drug contact, resulting in fast release within the first minutes of the test. Although the solvents used (PEG 400 and ethylene glycol diethyl ether) may introduce secondary solubilization effects, their contribution is limited compared to the impact of amorphization and wettability enhancement. The rapid and nearly complete dissolution observed shortly after contact with the medium supports the conclusion that the combined effects of amorphization, improved wettability, and enlarged surface area are primarily responsible for the dissolution improvement, whereas solubilization plays a supportive but not dominant role. An additional factor influencing the dissolution behavior of the LS systems was the order in which the carrier (microcrystalline cellulose) and the coating material (colloidal silica) were combined with the drug solution. The results showed that systems in which microcrystalline cellulose was added before colloidal silica exhibited faster dissolution, particularly when PEG 400 was used as the solvent. This can be explained by the fact that cellulose, due to its porous structure and larger absorption capacity, first captures and distributes the liquid drug solution within its internal pores. When silica is added subsequently, it covers the surface of cellulose particles and stabilizes the adsorbed liquid layer without significantly interfering with drug distribution. In contrast, adding silica first leads to partial adsorption of the liquid onto its much smaller surface area, limiting the penetration of the drug–solvent phase into the cellulose pores. As a result, the drug may be less uniformly dispersed, reducing the effective surface area upon contact with the dissolution medium. These observations indicate that the sequence of excipient incorporation affects the microstructure of the final LS powder, and its impact becomes more pronounced for solvents with higher viscosity or stronger interactions with the excipients, such as PEG 400.

In the broader context of solubility-enhancing technologies, the performance of the obtained LS systems is consistent with mechanisms reported for solid dispersions and nanosuspensions. Similar to amorphous solid dispersions, LS systems improve dissolution primarily through loss of crystallinity and enhanced wettability, which have been extensively described as key contributors to dissolution enhancement in BCS class II drugs (e.g., meloxicam and piroxicam solid dispersions) [3,28]. The amorphization-driven improvement observed in our study is also in line with reports demonstrating suppression of crystalline peaks and increased dissolution in LS systems of meloxicam and tadalafil [3,19]. In comparison with nanosuspensions, which rely mainly on particle size reduction to increase surface area and require stabilization of colloidal particles, LS systems offer a simpler and more robust manufacturing process. Nanosuspension technologies often demand high-energy milling and the use of surfactant-rich formulations, whereas LS systems enable molecular dispersion of the drug within a porous solid matrix without the need for intensive processing. The literature reports on nanosuspension-like systems prepared via LS technology, such as those for tadalafil and candesartan cilexetil [19,36], similarly attribute dissolution enhancement to improved surface area, dispersion, and wettability rather than particle size reduction alone. Thus, the LS approach used in this study combines the advantages of both solid dispersions (amorphization) and nanosized systems (enhanced interfacial surface), while remaining technologically simpler and more stable.

Although the present study focused on physicochemical characterization and dissolution profiling, further evaluation of the LS systems could include assays that provide additional predictive in vivo relevance. Because suvorexant is a BCS class II drug with high permeability, dissolution rather than permeability represents the primary rate-limiting step in absorption [24,25]. For this reason, dissolution testing is an appropriate first-line method for assessing formulation performance, and the improvements observed here are expected to translate into enhanced in vivo availability. Nevertheless, future work could expand the evaluation to biorelevant media (e.g., FaSSIF or FeSSIF) or in vitro permeability models to more closely mimic gastrointestinal conditions and further support in vivo predictability. Such studies fall beyond the scope of the current investigation, which was designed as a mechanistic analysis of LS system composition, amorphization behavior, and excipient interactions.

The influence of the order of excipient addition on dissolution can be explained by considering how LS systems are formed at the microscopic level. When microcrystalline cellulose is added first, it absorbs and distributes the liquid drug solution within its porous internal structure. Subsequent incorporation of colloidal silica then coats the cellulose particles, stabilizing the absorbed liquid phase and ensuring a more uniform dispersion of SUV within the matrix. In contrast, when silica is added before cellulose, a portion of the solvent may adsorb onto the silica surface, which has a much lower pore volume and different adsorption characteristics. This can reduce the penetration of the drug solution into the cellulose network and lead to less efficient distribution of SUV within the carrier. Although no morphological imaging was conducted in this study, this mechanistic interpretation aligns with widely reported LS system formation behavior and provides a plausible explanation for the observed differences in dissolution. We acknowledge that confirmation of microstructural differences would require dedicated imaging or spectroscopic methods, which fall outside the scope of this formulation-screening work.

Although parameters such as flowability, compressibility, and solid-state stability are essential when developing LS systems intended for tablet manufacturing, the present work was intentionally designed as an early-stage formulation screening. Only small-scale batches were prepared to evaluate key physicochemical characteristics and dissolution behavior, and at this stage, the powders were not targeted for direct compression nor produced in quantities sufficient for reliable flow or compressibility testing. Similarly, accelerated stability studies—including assessment of amorphous form recrystallization—require larger, optimized formulations and are typically conducted after lead compositions have been selected for further development. The LS systems investigated here, therefore, represent feasibility-oriented screening formulations, aimed at understanding solvent suitability and mechanistic effects of excipient ratios and order of addition. Comprehensive evaluation of flow characteristics, compressibility, and long-term or accelerated stability will be performed in future work when the most promising LS systems are advanced toward final dosage-form development.

## 4. Conclusions

Based on the analysis of the results obtained, the following conclusions can be formulated. Liquid–solid systems improve the dissolution rate of SUV. The amount and mutual ratio of excipients in LS systems is important because the greatest improvement in dissolution rate, regardless of the solvent used, was achieved when using the CCA variant of LS systems, containing microcrystalline cellulose and colloidal silica in a ratio of 10:1. The type of solvent used may affect the importance of the order in which auxiliary substances are added when preparing LS systems. When polyethylene glycol 400 was used as a solvent, this effect was observed, and when ethylene glycol diethyl ether was used as a solvent, no effect of the order of combining the ingredients was observed. Analyzing the physicochemical characteristics, it can be concluded that a loss of crystallinity of SUV was observed when it was introduced into liquid–solid systems. The physicochemical characterization demonstrated suppression of characteristic SUV crystalline signals in LS systems, suggesting its conversion into an amorphous or molecularly dispersed state. However, as no quantitative crystallinity analysis or spectroscopic evaluation was performed, these findings should be interpreted as indicative rather than definitive evidence of complete amorphization.

## Figures and Tables

**Table 1:** Composition of used ingredients for the preparation of liquid–solid systems.

Series	SUV (g)	Ethylene Glycol Diethyl Ether (g)	PEG 400 (g)	EMCOCEL M50 (g)	Aerosil (g)	EMOCEL:Aerosil Ratio	Mass of All Ingredients (g)
GCCA	0.0106	1.017	-	1.031	0.109	10:1	2.1676
GCCA2	0.0208	1.004	-	1.003	0.100		2.1278
PCCA	0.0101	-	1.006	1.002	0.102		2.1201
PCCA2	0.0208	-	1.003	1.000	0.101		2.1248
SCCA	0.0102	-	-	1.001	0.100		1.1112
GCA	0.0101	1.002	-	0.507	0.501	1:1	2.0201
GAC	0.0105	1.002	-	0.509	0.509		2.0305
PCA	0.0101	-	1.003	0.503	0.503		2.0191
PAC	0.0105	-	1.022	0.501	0.501		2.0345
GCA2	0.0205	1.002	-	0.502	0.502		2.0265
GAC2	0.0200	1.002	-	0.501	0.501		2.0240
PCA2	0.0203	-	1.003	0.509	0.502		2.0343
PAC2	0.0201	-	1.004	0.506	0.500		2.0301
SCA	0.0102	-	-	0.501	0.501		1.0122
SAC	0.0100	-	-	0.501	0.501		1.0120

**Table 2:** Similarity coefficient f_2_ for release profiles of LS systems vs. series without solvent.

Compared Series				
LS series		Series Without Solvent		
		SCCA	SCA	SAC
CCA	GCCA	24.88	-	-
	PCCA	30.54	-	-
CA	GCA	-	40.78	-
	PCA	-	30.67	-
AC	GAC	-	-	34.41
	PAC	-	-	45.60

## Data Availability

The raw data supporting the conclusions of this article will be made available by the authors on request.

## Supplementary Materials

The following supporting information can be downloaded at: https://www.mdpi.com/article/10.3390/polym18080936/s1, Table S1: Similarity coefficients f2 for release profiles of compared series.
