# Supplementary material for: The Influence of Liquid–Solid Preparations on the Dissolution of Suvorexant
Source: Polymers (Basel). 2026 Apr 10;18(8):936. doi: 10.3390/polym18080936 (PMC13119769; doi:10.3390/polym18080936)
Supplement: Supplementary file 1 [file polymers-18-00936-s001.zip › polymers-4155072-supplementary.pdf]

Table S1 Similarity coefficients f_2 for release profiles of compared series

compared LS	GCCA2	GCA	GCA2	GAC	GAC2	PCCA	PCCA2	PCA	PCA2	PAC	PAC2	SCCA	SCA	SAC
GCCA	69.66	50.15	77.58	46.49	92.01	52.96	61.26	56.92	39.16	29.58	36.57	24.88		
GCCA2		58.67	68.06	54.03	65.77	63.82	78.03	49.84	44.17	32.86	41.30			
GCA			52.59	77.57	48.16	81.07	66.64	39.05	50.25	38.50	49.85		40.78	
GCA2				47.92	73.83	54.75	62.04	50.90	38.76	29.79	36.89			
GAC					44.84	73.73	60.99	37.25	54.86	41.42	55.63			34.41
GAC2						50.84	58.24	59.36	38.05	28.83	35.54			
PCCA							75.54	41.10	50.44	37.61	49.06	30.54		
PCCA2								45.68	47.32	34.96	44.61			
PCA									34.05	25.73	31.16		30.67	
PCA2										49.83	65.61			
PAC											55.34			45.60
